# Influence of the Vehicle on the Penetration of Particles into Hair Follicles

**DOI:** 10.3390/pharmaceutics3020307

**Published:** 2011-06-14

**Authors:** Alexa Patzelt, Heike Richter, Lars Dähne, Peter Walden, Karl-Heinz Wiesmüller, Ute Wank, Wolfram Sterry, Jürgen Lademann

**Affiliations:** 1 Center of Experimental and Applied Cutaneous Physiology (CCP), Department of Dermatology and Allergology, Charité – Universitätsmedizin Berlin, 10117 Berlin, Germany; E-Mails: heike.richter@charite.de (H.R.); wolfram.sterry@charite.de (W.S.); juergen.lademann@charite.de (J.L.); 2 Surflay Nanotec GmbH, Schwarzschildstr. 8, 12489 Berlin, Germany; E-Mail: l.daehne@surflay.com; 3 Department of Dermatology and Allergology, Charité – Universitätsmedizin Berlin, Tumorimmunology, 10117 Berlin, Germany; E-Mail: peter.walden@charite.de; 4 EMC microcollections GmbH, Sindelfinger Str. 3, 72070 Tübingen, Germany; E-Mails: wiesmueller@microcollections.de (K.-H.W.); wank@microcollections.de (U.W.)

**Keywords:** hair follicle, penetration, particle, vehicle, formulation

## Abstract

Recently, it has been demonstrated that particulate substances penetrate preferentially into the hair follicles and that the penetration depth depends on the particle size. In the present study, the influence of the vehicle of the particulate substances on the penetration depth was investigated. Four different formulations (ethanolic suspension, aqueous suspension, ethanolic gel and aqueous gel) containing peptide-loaded particles of 1 μm in diameter were prepared and applied on porcine ear skin. After penetration, punch biopsies were taken and the penetration depths of the particles were investigated by laser scanning microscopy. The deepest penetration was achieved with the gel formulations demonstrating an influence of the vehicle on the penetration depth of particulate substances.

## 1. Introduction

Particulate substances are increasingly utilized as carriers for active ingredients in dermatological and cosmetic products to improve efficacy of delivery. Whereas, intercellular penetration of particulate substances via the lipid layers of the stratum corneum can be considered implausible because of size, the hair follicles have been demonstrated to be the main target for topically applied particles in undamaged skin [1]. Hair follicles are breaks in the otherwise highly efficient skin barrier and, therefore, act as pit traps for topically applied particles [2]. Effective delivery of active agents, however, is also strongly related to its efficient release from the delivering particles subsequent to the transport into the hair follicle allowing their independent penetration through the skin barrier into the living tissue and, if desired, their diffusion to the target structures. Several release mechanisms are being explored such as external stimulation or enzymatic release [3].

The hair follicle itself can be divided into at least four target areas for topical application: the sebaceous gland, the bulge region, the hair matrix cells and the hair follicle infundibulum [4]. The latter provides an interrupted barrier with increased permeability and is surrounded by a high density of immune cells and an extensive capillary network important for penetration and systemic absorption of active agents [5,6].

Recently, it could be demonstrated that selective delivery of particulate substances to the desired target sites can be realized by modification of the particle size [2]. Deepest penetration was found for particles between 300 and 600 nm, which corresponds to the thickness of the keratin scales of the hair [7]. For smaller and larger particles significantly lower penetration depths were determined. Based on these findings it was hypothesized that the moving hair and the hair follicle might act as an active transport system shuffling the particles into the hair follicles.

Moreover, it is established that efficacy, tolerability and application properties of topically applied products are clearly related to the type of vehicle used [8]. The variety of vehicles available can be distinguished by their galenic properties such as rheological properties, polarity and physicochemical features according to the guideline of the European Pharmacopoeia [8], all having an impact on the penetration properties of substances. Aqueous suspension is considered to be the standard vehicle, whereas ethanol is known as a penetration enhancer through extracting the lipids from the stratum corneum [8]. Also, hydrophilic gel preparations are known to improve drug delivery of substances into the skin [9].

Therefore, the aim of the present study was to investigate the influence of the vehicle on the penetration of SiO_2_ particles reversibly loaded with a model peptide. For this purpose, four different vehicles were selected: an aqueous and an ethanolic suspension, an aqueous agarose gel and an ethanolic gel. For identification and tracing, the particles and the peptide were labeled with different fluorescence dyes. The investigations were performed on porcine skin as a model for human skin [10,11]. After application and penetration, follicular penetration depths of the particles and the loaded model peptide were investigated.

## 2. Experimental Section

### 2.1. Skin samples

The porcine ears were obtained immediately after slaughtering from the butcher. Pre-treatment of the porcine ear skin included rinsing with cold water, drying with paper towels and carefully shortening of the bristles. Approval for these experiments had been obtained from the Government Office of Veterinary Medicine in Berlin-Treptow, Germany.

### 2.2. Particle preparation and formulations

For reversible coupling of the peptide, monodisperse silica particles of 1 μm in diameter (FUSO, Japan) were coated by the Layer-by-Layer® process [12] with alternating layers of the polycation poly(allylamine hydrochloride) PAH and the polyanions poly(styrene sulfonate) PSS and poly(methacrylic acid sodium salt) PMAA. All polyelectrolyte solutions were prepared as 1 g/L polymer, 200 mM salt and 50 mM acetate buffer pH 5.6 in MilliQ water. The coating sequence was silica/PAH/PSS/(PAH-Cy5/PSS)_2_/PAH/PMAA. For the third and fifth layers, PAH covalently linked at every 180th monomer unit with the fluorescence dye Cy5 was used. Thickness of the polymer layers was around 20 nm in sum as reported in several publications [12]. The polymer-coated particles were carefully washed with water and the pH was set to 8 by addition of 50 mM Tris-buffer in order to load them with the peptides.

A synthetic peptide modified by a positively charged amino acid tag and labeled with rhodamine B (6 mg) was added overnight to the suspension of polymer-coated particles (100 mg) for adsorption to the negatively charged polymer surface of the particles. After incubation, excess of peptide was washed off. The amount of peptide loaded on the particles was determined by UV/Vis spectroscopy (λ_abs (Cy5)_ = 643 nm) of the collected supernatants as 41 μg peptide/mg particles.

The peptide-loaded particles were diluted to a 1% suspension in 10 mL Tris-buffer and divided into 4 aliquots. The suspensions were centrifuged, the supernatants removed and the deposited particles thoroughly mixed with 2.5 mL of the following media for a 1% suspension resp. gel-mixture:

- MilliQ water
- 20% ethanol in water
- low melting agarose gel (45 °C) at 0.5% in the liquid state
- low melting ethanolic gel (Actensa, Berlin, Germany)

For preparation of the aqueous gel, 0.5% Agarose (m.p. 45 °C) was dissolved in water at 60 °C. After cooling down to 45 °C, a concentrated particle suspension (10%) in water was mixed with the gel at a ratio of 1:10. The ethanolic gel contains 80% ethanol, water and gel constituents in the 1–2% range and could be used as is.

### 2.3. Application protocol

The experiments were performed on six porcine ears in parallel. Preparation of the skin included marking of 4 areas A–D of 2 × 3 cm in size (one for each formulation) on each porcine ear and the application of a silicone barrier around the marked skin areas in order to avoid lateral spreading. Subsequently, 120 μL of each of the 4 formulations were applied homogeneously onto the skin areas A–D using a massage appliance for 3 minutes (Massage Gerät PC60, Petra electric, Burgau, Germany). After a penetration time of one hour, punch biopsies of 3 mm in diameter were collected from the porcine tissue. The samples were shock-frozen in liquid nitrogen and longitudinal cryo sections of 10 μm thickness were prepared using the cryotome HM 560 Cryo-Star, Microm International GmbH, Walldorf, Germany.

### 2.4. Sample analysis

The sections were analyzed using a laser scanning microscope LSM 2000 (Carl Zeiss, Jena, Germany). To avoid double excitation of both dyes, the respective excitation wavelengths had to be shifted out of the absorption maximum. Therefore, rhodamine was excited at 488 nm and Cy5 at 568 nm. Emission was measured at 515 nm for rhodamine and 665 nm for Cy5, respectively, for identification of the stained particles and peptides. Intensities were not used for evaluation.

**Figure 1.**
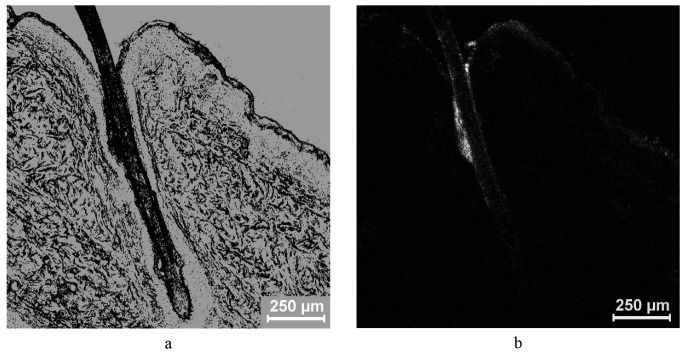
Images of a longitudinal section of a hair follicle taken in the transmission (**a**) and the fluorescence mode (**b**) by laser scanning microscopy.

The same samples were additionally imaged in transmission mode in order to identify the follicular structure. By superposition of the images from both visualization modes, the maximal penetration depths of the fluorescent particles and of the fluorescent peptide were determined. In Figure 1, a longitudinal section of a hair follicle taken in the transmission and the fluorescent mode is exemplarily depicted.

### 2.5. Statistical analysis

For statistical analysis, the distances covered by the deepest penetrating fluorescent particles resp. peptide molecules were measured. Mean values and standard deviations were calculated from different hair follicles and compared using Microsoft Excel®. Further statistical analysis was done with the software program SPSS® 16.0. To compare the follicular penetration depths of the different particle preparations, the t-test was utilized at a significance level of p < 0.05.

## 3. Results and Discussion

**Figure 2.**
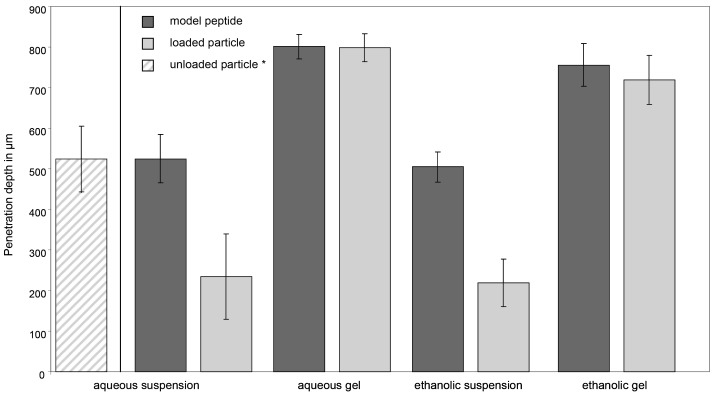
Follicular penetration depths of the loaded particles and the model peptide applied in four different vehicles. (* penetration depth of unloaded bare SiO_2_ particles in aqueous suspension for comparison, data utilized from previous study [2]).

Polymer coated SiO_2_ particles labeled with Cy5 were reversibly loaded with a rhodamine-labeled peptide. These peptide-loaded particles were formulated with four different vehicles. The penetration depths of the particles and the loaded peptide in the porcine ear skin hair follicles were investigated separately and are summarized in Figure 2. The results revealed differences in the penetration depths with the different formulations as well as between the particles and peptide.

The penetration depths of the particles and peptide were significantly deeper (p < 0.05) with the gel preparations than with the suspensions. Whereas with the gel formulations comparable penetration depths were observed for particles and peptide (p > 0.05), with suspensions the peptide penetrated significantly deeper into the hair follicles than the particles (p < 0.05). No differences were observed between ethanolic and aqueous formulations.

The penetration efficacy of topically applied substances potentially depends on the properties of the active agent, the carrier and the vehicle in combination. While previous investigations showed that the penetration of bare SiO_2_ particles mainly depends on particle size when applied in aqueous suspension [2], the present study demonstrates that in addition the vehicle, *i.e.*, formulation, influences the penetration of particulate substances.

With the gel preparations, significantly increased penetration depths could be achieved when compared to the suspensions. This suggests that aggregation tendency of the particles might be reduced in a viscous gel preparation by stabilizing a homogeneous dispersion of carriers in the matrix as described in literature [9].

Related observations have been reported by other investigators. Hydrophilic gels were shown to serve as effective and inert environments for carriers including nanocapsules [13], liposomes [14], solid lipid nanoparticles [15] and microemulsions [16]. Sintov and colleagues [17] performed an *in vitro* diffusion study of flutamide gel combined with a penetration enhancer. They demonstrated the beneficial effect of the gel in comparison to a hydroalcoholic solution or a gel containing no penetration enhancer.

The release of the peptide from the delivering particles could clearly be demonstrated. In aqueous and ethanolic suspensions, the peptide penetrates significantly deeper into the follicles than the particles. Efficient release of the peptide triggered by the physiological conditions in the hair follicles and its independent diffusion responsible for this outcome. The release effect was less impressive for the gel preparations and especially for the aqueous gel. Using the ethanolic gel preparation, the peptide also penetrated slightly deeper into the hair follicles than the delivering particles, although here the differences were not significant (p > 0.05).

Compared to our previous study, the peptide-loaded particles applied in aqueous suspension penetrated less far into the hair follicles than the bare SiO_2_ particles (234 ± 105 *versus* 524 ± 81 μm). As the application protocol, study conductor, skin model and particle preparation were identical, loading of the particles with the model peptide seems to have an effect. The increased size of the particles due to the coating (40 nm increase in diameter in the dry state but up to 100 nm when swollen in the suspension), and also the state of aggregation, because of the physico-chemical properties of the peptide, may affect the penetration of the particles.

The present study revealed no penetration-enhancing effect of ethanol, neither for the suspension and nor for the gel formulations. Even though ethanol is described as classical penetration enhancer for intercellular penetration, explained with the extraction of lipids from the stratum corneum [8], this might not be applicable to follicular penetration processes. This observation supports our hypothesis of a simple mechanical mechanism for particle transport into the hair follicles.

## 4. Conclusion

In conclusion, in addition to the particle size, the loading and the vehicle can also significantly impact the follicular penetration depth of particulate substances. This effect may be related to altered aggregation states of the particles which may indirectly influence particle size and the efficiency of the mechanical transport mechanism.

## References

[1] Lademann J., Richter H., Teichmann A., Otberg N., Blume-Peytavi U., Luengo J., Weiss B., Schaefer U.F., Lehr C.M., Wepf R., Sterry W. (2007). Nanoparticles - an efficient carrier for drug delivery into the hair follicles. Eur. J. Pharm. Biopharm..

[2] Patzelt A., Richter H., Knorr F., Schaefer U., Lehr C.M., Daehne L., Sterry W., Lademann J. (2011). Selective follicular targeting by modification of the particle sizes. J. Control. Release.

[3] Mak W.C.R.H., Patzelt A., Sterry W., Lai K.K., Renneberg R., Lademann J. (2011). Drug delivery into the skin by degradable particles. Eur. J. Pharm. Biopharm..

[4] Patzelt A., Knorr F., Blume-Peytavi U., Sterry W., Lademann J. (2008). Hair follicles, their disorders and their opportunities. Drug Discov. Today: Dis. Mech..

[5] Vogt A., Blume-Peytavi U. (2003). Biology of the human hair follicle. New knowledge and the clinical significance. Hautarzt.

[6] Vogt A., Mandt N., Lademann J., Schaefer U., Blume-Peytavi U. (2005). Follicular targeting - a promising tool in selective dermatotherapy. J. Investig. Dermatol. Symp. Proc..

[7] Lademann J., Patzelt A., Richter H., Antoniou C., Sterry W., Knorr F. (2009). Determination of the cuticula thickness of human and porcine hairs and their potential influence on the penetration of nanoparticles into the hair follicles. J. Biomed. Opt..

[8] Daniels R., Knie U. (2007). Galenics of dermal products - vehicles, properties and drug release. J. Dtsch Dermatol. Ges..

[9] Batheja P., Sheihet L., Kohn J., Singer A.J., Michniak-Kohn B. (2011). Topical drug delivery by a polymeric nanosphere gel: Formulation optimization and in vitro and in vivo skin distribution studies. J. Control. Release.

[10] Jacobi U., Kaiser M., Toll R., Mangelsdorf S., Audring H., Otberg N., Sterry W., Lademann J. (2007). Porcine ear skin: an *in vitro* model for human skin. Skin Res. Technol..

[11] Meyer W., Schwarz R., Neurand K. (1978). The skin of domestic mammals as a model for the human skin, with special reference to the domestic pig. Curr. Probl. Dermatol..

[12] Peyratout C.S., Daehne L. (2004). Tailor-made polyelectrolyte microcapsules: from multilayers to smart containers. Angew. Chem. Int. Ed. Engl..

[13] Alvarez-Roman R., Barre G., Guy R.H., Fessi H. (2001). Biodegradable polymer nanocapsules containing a sunscreen agent: preparation and photoprotection. Eur. J. Pharm. Biopharm..

[14] Glavas-Dodov M., Goracinova K., Mladenovska K., Fredro-Kumbaradzi E. (2002). Release profile of lidocaine HCl from topical liposomal gel formulation. Int. J. Pharm..

[15] Pople P.V., Singh K.K. (2006). Development and evaluation of topical formulation containing solid lipid nanoparticles of vitamin A. AAPS Pharm. Sci. Tech..

[16] Chen H., Chang X., Du D., Li J., Xu H., Yang X. (2006). Microemulsion-based hydrogel formulation of ibuprofen for topical delivery. Int. J. Pharm..

[17] Sintov A., Serafimovich S., Gilhar A. (2000). New topical antiandrogenic formulations can stimulate hair growth in human bald scalp grafted onto mice. Int. J. Pharm..

